# Aligning cellulose nanofibril dispersions for tougher fibers

**DOI:** 10.1038/s41598-017-12107-x

**Published:** 2017-09-19

**Authors:** Pezhman Mohammadi, Matti S. Toivonen, Olli Ikkala, Wolfgang Wagermaier, Markus B. Linder

**Affiliations:** 1 Department of Bioproducts and Biosystems, School of Chemical Engineering, Aalto University, P.O. Box 16100, FI-16100 Espoo, Finland; 2 Department of Applied Physics, School of Science, Aalto University, P.O. Box 15100, FI-00076 Espoo, Finland; 3 Department of Biomaterials, Max Planck Institute of Colloids and Interfaces, D-14424 Potsdam, Germany

## Abstract

Nanocomposite materials made from cellulose show a great potential as future high-performance and sustainable materials. We show how high aspect ratio cellulose nanofibrils can be efficiently aligned in extrusion to fibers, leading to increased modulus of toughness (area under the stress-strain curve), Young’s modulus, and yield strength by increasing the extrusion capillary length, decreasing its diameter, and increasing the flow rate. The materials showed significant property combinations, manifesting as high modulus of toughness (~28–31 MJ/m^3^) vs. high stiffness (~19–20 GPa), and vs. high yield strength (~130–150 MPa). Wide angle X-ray scattering confirmed that the enhanced mechanical properties directly correlated with increased alignment. The achieved moduli of toughness are approximately double or more when compared to values reported in the literature for corresponding strength and stiffness. Our results highlight a possibly general pathway that can be integrated to gel-spinning process, suggesting the hypothesis that that high stiffness, strength and toughness can be achieved simultaneously, if the alignment is induced while the CNF are in the free-flowing state during the extrusion step by shear at relatively low concentration and in pure water, after which they can be coagulated.

## Introduction

Structural biological materials have received extensive attention in materials science due to their impr**e**ssive mechanical properties achieved with *in-vivo* processes in ambient conditions^1^. One such desirable function is to combine strength, stiffness and toughness^2–6^. Such properties in materials typically arise from their structure^1,7^, often involving an intricate hierarchical architecture utilizing stiff and strong reinforcing elements embedded in a softer, energy dissipating matrix^2–6^. Much studied examples of such materials are bone, nacre, and cellulose in plant cell walls^7^. These materials have inspired researchers to fabricate analogous architectures seeking similar composite properties^1^. However, the combination of toughness with stiffness and strength has remained elusive for most man-made materials as strength and stiffness often lead to brittleness. The mechanisms of combining strength and toughness remains a challenge for materials science^8^.

Cellulose nanofibrils (CNF, also called cellulose nanofibers, nanofibrillated cellulose, NFC, or microfibrillated cellulose, MFC), have drawn abundant attention due to an attractive combination of properties, namely excellent mechanical properties (i.e. elastic modulus of 29–36 GPa^9^ and proposed tensile strength of 1–3 GPa^10,11^ along the fibril axis for individual fibrils) combined with relatively low density (~1.5 g/cm^3^), the possibility of extraction from various, widely available, and renewable resources^12–14^, and promising biocompatibility^15^. These features together make CNF an attractive raw-material candidate also for spinning the next generation environmentally friendly fibers^16–19^.

However, the CNF-based materials thus far reported are still not achieving the full mechanical potential of the constituent components. One reason for this is presumably a lack of the sufficient nanofibril alignment, which is a challenge that has drawn much attention^16,20,21^. Significant increases in stiffness and strength have been reported, but these have come at the expense of reduced extensibility, with most aligned CNF-based materials encountering the generally acknowledged challenges of combining strength/stiffness with toughness^8^. Therefore, in addition to the observed components in biological materials, how they are processed should also be considered, as the processing of a material and its final properties are tightly connected. A theme that is rarely addressed is that the observed deterioration of the extensibility upon increased alignment might actually be due to generation of structural defects, such as loosely bound aggregates or poorly connected regions in the network, during processing. Under this hypothesis, engineering of a processing pathway leading to reduced amount and sizes of structural defects could lead to material properties closer to those of the constituent fibrils. For this purpose, inspiration can be taken from the structure of the high aspect ratio spinning duct of orb-weaving spiders for fiber spinning of colloidal materials^22–24^. Several central parameters are specific to the spider silk proteins but also more general guidelines can be extracted. For one, the aggregation of the colloidal dispersion is delayed until the material is already highly aligned by the shear forces^25–28^. Secondly, the total amount of shear is amplified by the high aspect ratio of the spinning duct, which may be up to centimeters in length and down to micrometers in inner diameter^29,30^.

This suggests to study flow-alignment of CNF nanofibrils in a non-aggregated state, in contrast to processing routes that, prior to or during the alignment, lock the structures by promoting interactions between the nanofibrils by coagulation and ionic strength changes^16,20,21^. Consequently, in this work we study how free-flowing CNF fibrils can be aligned in aqueous conditions and relatively low concentration using high flow conditions in high aspect ratio extrusion capillaries. We investigate systematically the parameters for the wet-spinning and spinning path geometries and show how they improve the alignment and mechanical properties of the spun fibers.

## Results and Discussion

**Figure 1:**
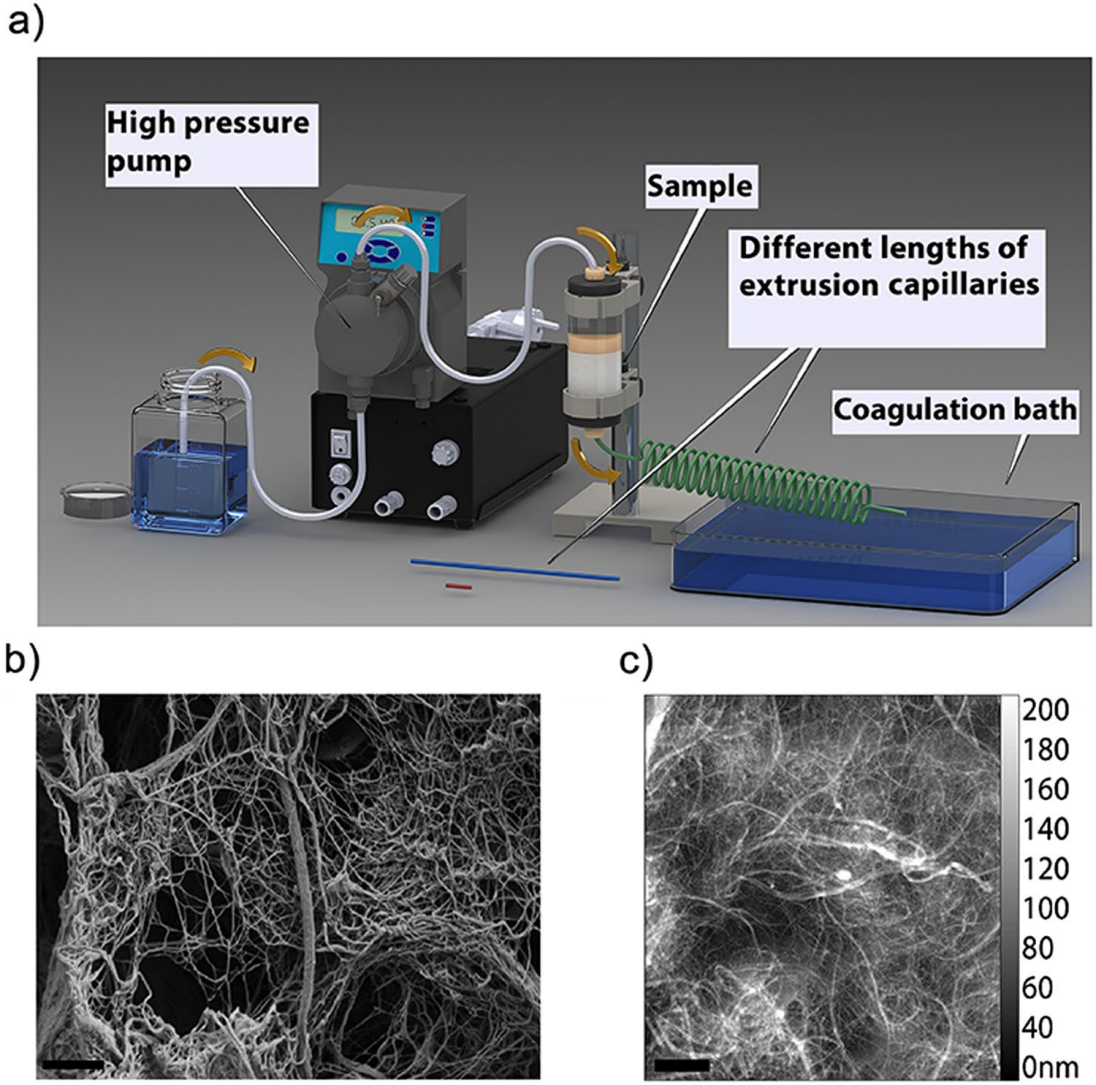
The scheme of the spinning device and SEM and AFM images of the starting CNF. (**a**) The capillary length, the diameter, and the flow rate were modified, (**b**) SEM high magnification image of cellulose nanofibrils (CNF), (**c**) tapping mode AFM. The morphology and size distribution of the CNF were characterized by AFM and show fibrils with diameters in the range of 5 to 500 nm and lengths up to several micrometers since the ends of fibrils were not recognizable in the AFM topography images. Scale bars are 1 μm in both images.

Nanocellulose fibers were made by extruding an aqueous dispersion of CNF through an extrusion capillary into a coagulation bath using the setup illustrated in Fig. 1a. SEM and AFM micrographs of the fibrils used are shown in Fig. 1b,c. The main factors affecting the fiber properties were the length and the inner diameter of the extrusion capillaries, and the flow rate of the CNF dispersion inside the capillary. The flow properties and aggregation behavior of CNF depend heavily on its concentration (Supplementary Fig. S1 and Table S1). After an initial screening, the concentration of 2% w/v was selected for further investigations to explore the effect of flow properties to mechanical properties. As expected, the CNF dispersions showed shear thinning behavior at all screened concentrations (Supplementary Fig. S2).

**Figure 2:**
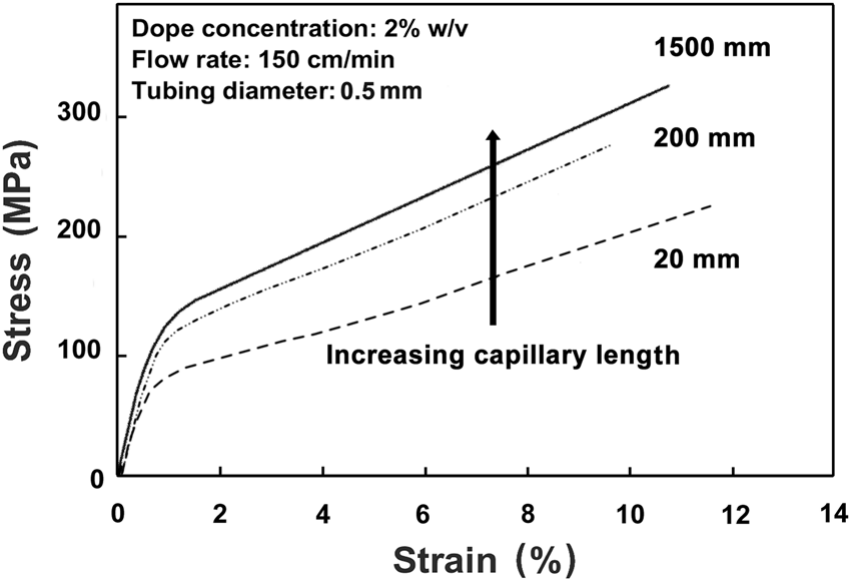
Representative stress-strain curves of fibers spun using different capillary lengths. Capillary lengths: 1500 (solid), 200 (dot-dash), and 20 mm (dash). Other fiber spinning parameters were kept constant: the capillary inner diameter was 0.5 mm, CNF concentration was 2% w/v, and the flow rate was 150 cm/min.

The achieved mechanical properties improved significantly by increasing the extrusion capillary length and decreasing the inner diameter, as well as by increasing the flow rate in the capillary. These effects were seen independently for each parameter, but also synergistically. Figure 2 shows representative stress-strain curves for fibers prepared with varying capillary lengths (20, 200, and 1500 mm) while the other parameters were kept constant (see more details in Supplementary Fig. S3). Clear increases in the Young’s modulus (12.9 to 18.8 GPa, i.e., increase by 46%), yield strength (91 to 139 MPa, + 53%), and ultimate tensile strength (265 to 328 MPa, + 24%) can be seen upon increasing the capillary length from 20 to 1500 mm, while notably the ultimate strain was unaffected and remained high (11.6 to 12.1%). The preservation of a high ultimate strain upon enhancement of strength and stiffness is of great interest as it is in stark contrast to what is reported in the related literature^16–18,20,21,31,32^. Similar improvements on mechanical properties are seen upon increase of flow rate and decrease of inner diameter of the extrusion capillary (Supplementary Figs S4 and S5, and Table S2). Variation and scatter of the mechanical properties in relation to spinning parameters are summarized in Supplementary Fig. S6. In the following discussion, the main focus is on the effect of the capillary length, as it provided a straightforward route to systematically alter mechanical properties.

**Figure 3:**
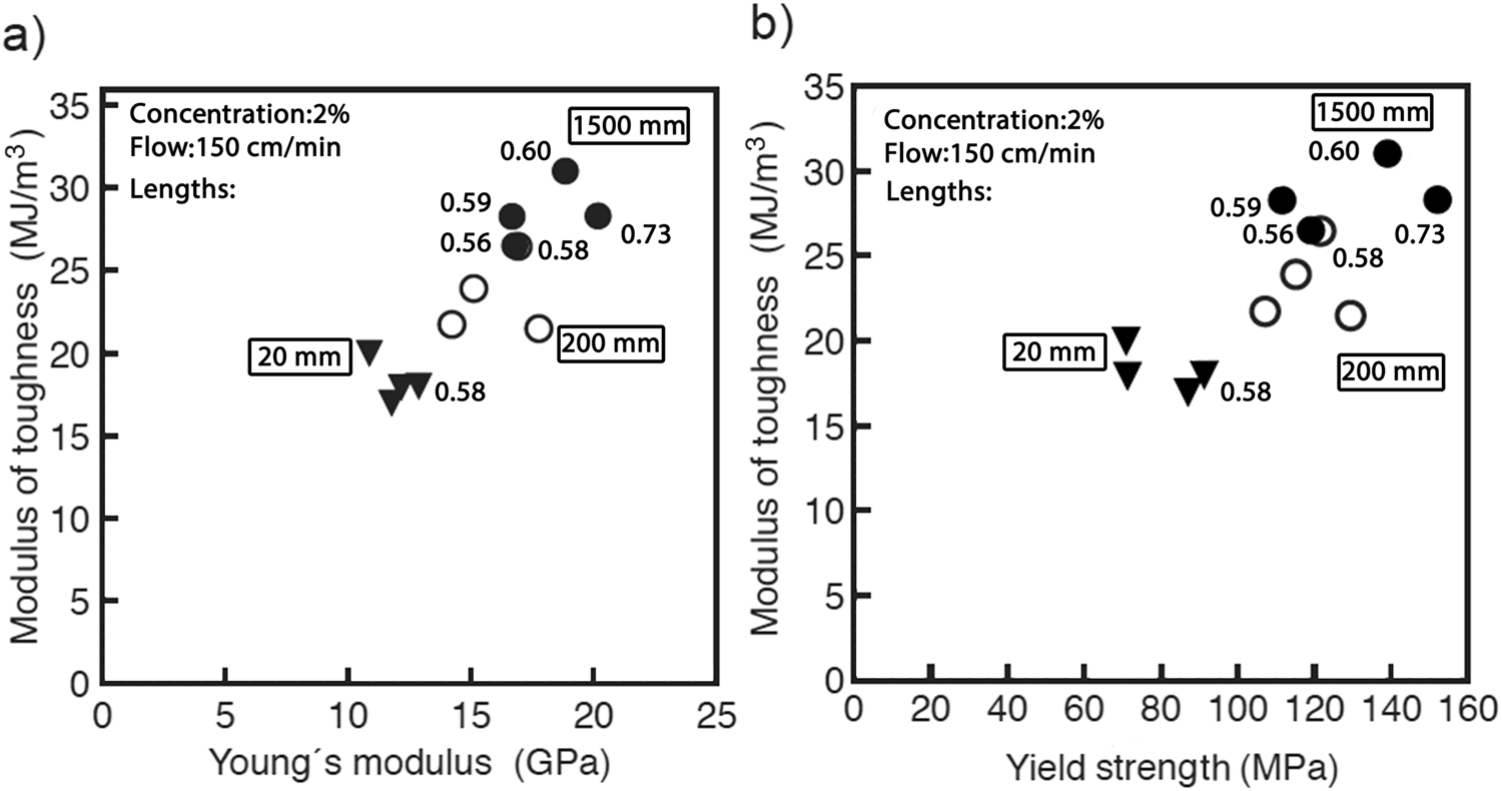
The modulus of toughness vs. Young’s modulus (**a**) and yield strength (**b**) of the CNF fibers. Fibers spun by extruding the CNF dispersion through longer capillaries exhibit higher mechanical properties. Capillary lengths were 20 mm (filled inverted triangles), 200 mm 5 (empty circles), and 1500 mm (filled circles). The modulus of toughness was calculated from the area under the stress-strain curve. Plotting the modulus of toughness vs. either Young’s modulus (**a**), or yield strength (**b**) revealed a nearly linear positive correlation. Values next to selected data points denote the Hermans orientation parameters obtained for the corresponding fibers with WAXS (see below).

The consequence of elevated strength and stiffness, while maintaining high extensibility, is that the area under the stress-strain curve was greatly increased. Integrating the area under the stress-strain curve results in a measure of toughness of the material and is known as the modulus of toughness^33–35^. It describes the energy absorbed by the material per unit volume upon deformation until fracture^36–38^. Surprisingly, plotting the modulus of toughness with respect to Young’s modulus or yield strength shows a nearly linear correlation (Fig. 3). This finding of positive correlation between toughness and stiffness or strength is significant because in man-made materials there tends to be a mutually exclusive tradeoff where increasing one reduces the other^8,38–40^.

**Figure 4:**
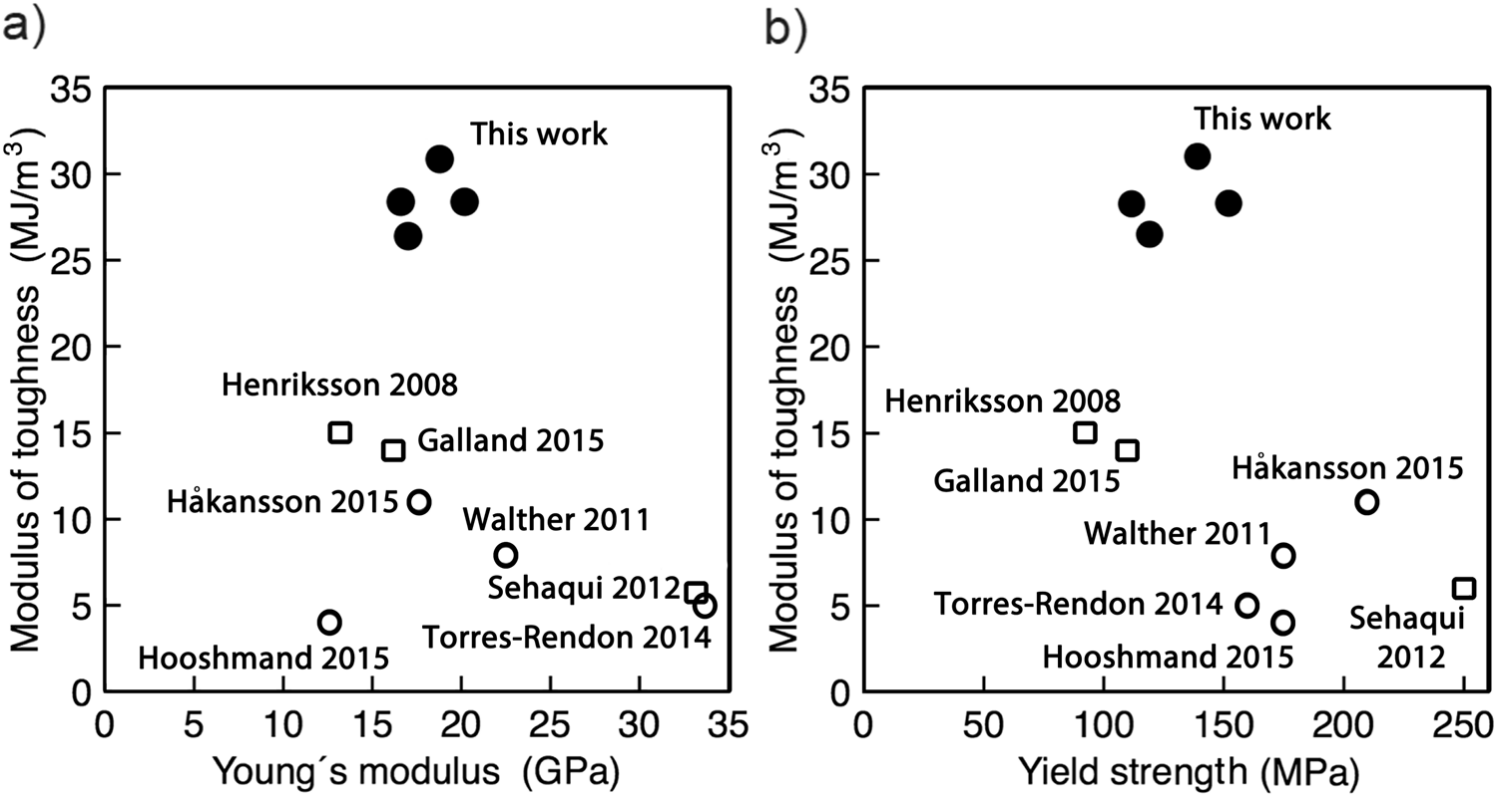
Modulus of toughness vs. Young’s modulus (**a**) and yield strength (**b**) for CNF fibers produced in this work (filled circles) in comparison to those reported in existing literature (empty symbols). In this work (filled circles) a modulus of toughness is observed at least twice as high as compared to films (empty squares) and fibers (empty circles) reported in the literature^16,17,19–21,31,32^. The four data points shown from this work are from fibers produced using 2% w/v CNF concentration, flow rate 150 cm/min and 1500 mm capillary but varying the inner diameter (0.4, 0.5, 0.75 and 1 mm).

To place our findings in perspective, we plotted the modulus of toughness against both Young’s modulus and yield strength together with previously reported values for fibers and films in comparable studies (Fig. 4). We noted that the present materials showed a modulus of toughness values of about double compared to those of CNF for the same Young’s modulus or yield strength values (Supplementary Table S3)^16,17,19–21,31,32^.

**Figure 5:**
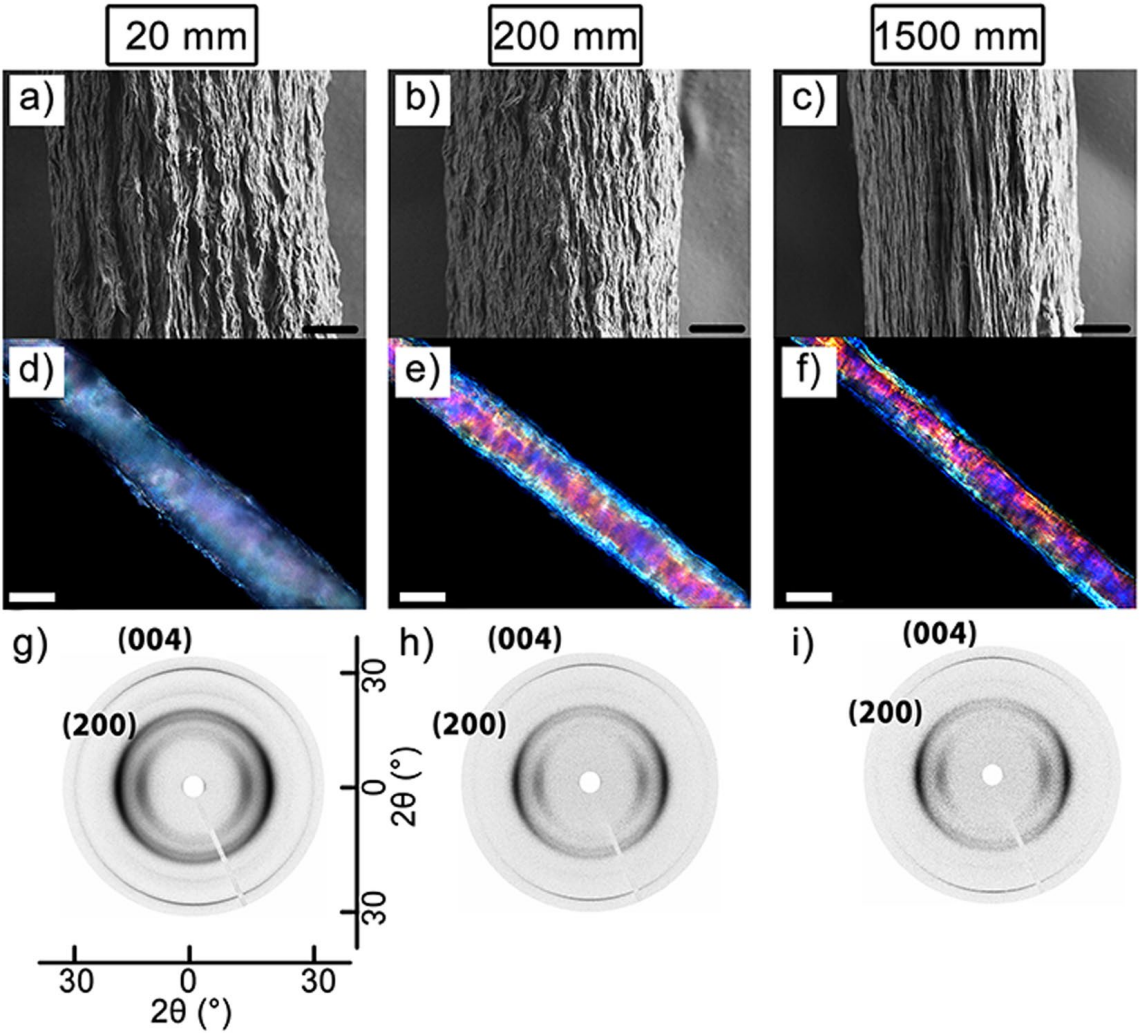
Effect of the length of extrusion capillary on the alignment. The left, middle and right columns show data for fibers made with capillaries of 20, 200 and 1500 mm length respectively. SEM micrographs (**a**,**b**,**c**) represent the surface topology of the fibers showing and increasing smoothness/orientation of surface structures with capillary length. Optical birefringence of same fibers illustrating pronounced changes in the birefringence (**d**,**e**,**f**). Polarized optical microscopy images of corresponding CNF fibers showing increased birefringence for samples made with longer capillaries. WAXS diffractograms (**g**,**h**,**i**) of single fibers indicating increasing alignment with increasing capillary length. In all cases the capillary had an inner diameter of 0.5 mm, the CNF concentration was 2 % w/v, and the linear flow rate was 150 cm/min. Scale bars are 10 μm in (**a,b**, and **c**), and 50 μm in (**d,e,** and **f**).

Alignment of the CNF along the longitudal axis of the fiber (see Fig. 5) was explored for understanding mechanisms of the high mechanical properties. The SEM images in Fig. 5a,b,c show both preferential alignment of the surface texture of the fibers, as well as densification of the fibers seen as a reduction in diameter (see also Table S2), when the length of the extrusion capillary is increased. Similarly, the alignment of intrinsically birefringent CNF^41^ leads to an increase in birefringence of the fibers, and hence more intense colors are observed (Fig. 5d–f) when the fibers are imaged between crossed polarizers. The observed colors and their intensities are a product of both the thickness and the birefringence of the sample at each point^42^, explaining the variation of color observed across the fiber. The anisotropy can also be seen qualitatively in diffractograms obtained by wide angle X-ray scattering (WAXS) (Fig. 5g,h,i).The data show that the fiber produced by the longest capillary shows the highest alignment in the axial direction. SEM imaging of the cross-sectional structure of the fibers exposed by cryofracturing (Supplementary Fig. S7) further supports an increased degree of CNF alignment with increased extrusion capillary length. Fracture surfaces after tensile testing are shown in Supplementary Fig. S8.

Hermans orientation parameters and orientation indices were calculated from the azimuthal intensity profile of the (004) reflection in the WAXS diffractograms. Values of the Hermans orientation parameter and the orientation index varied from 0.52 to 0.61 and from 0.706 to 0.794, respectively (Supplementary Table S4). The fibers produced with the longest (1500 mm) and thinnest (0.4 mm) capillary with high flow rate (150 cm/min) displayed the highest Hermans orientation parameter of 0.61, whereas the fibers extruded with the shortest capillary (20 mm) or a low flow rate (15 cm/min) showed a lower Hermans orientation parameter (0.56). The lowest Hermans orientation parameter (0.52) was found for the fiber produced with high CNF concentration (4%).

**Figure 6:**
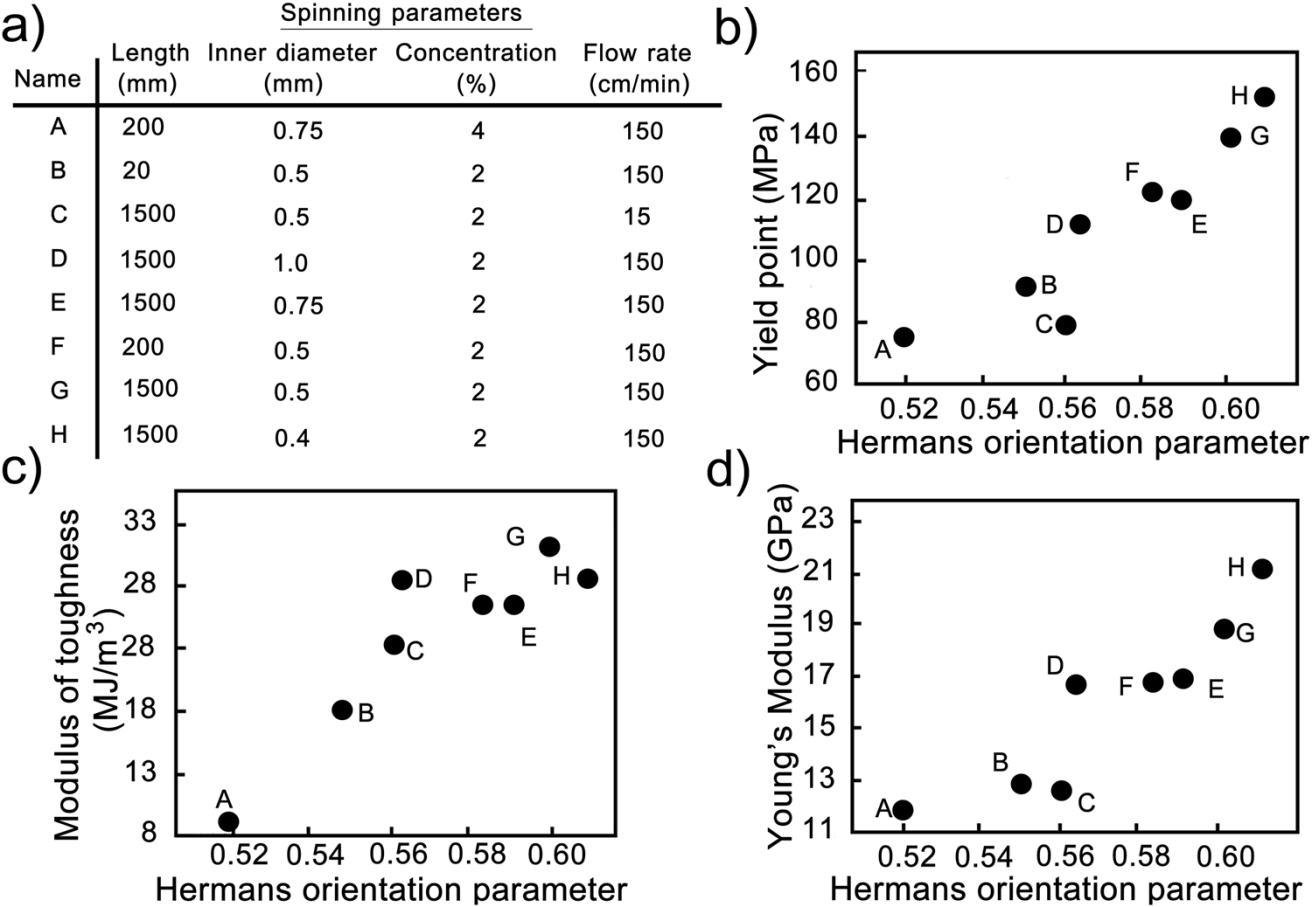
A comprehensive correlation between CNF alignment and mechanical properties. (**a**) List of samples with one-letter codes used to identify samples in the plots. Hermans orientation parameter for (004) reflection correlated to (**b**) yield strength, (**c**) stiffness and (**d**) modulus of toughness for the fibers spun with different lengths and inner diameters of extrusion capillaries, and at high and low flow rates, and with different concentrations of CNF dispersion. Numeric values are listed in in Supplementary Table 4. Corresponding plots for ultimate strain, ultimate tensile strength and slope of the stress-strain curve after the yield point are in Supplementary Fig. S9.

In Fig. 6 we show Hermans orientation parameters plotted against the modulus of toughness, yield point, and stiffness (see Supplementary S9 for corresponding plots for the ultimate strain, the tensile strength, and the slope of the stress-strain curve after the yield point). There was a strong correlation between the orientation parameter and the mechanical properties. Comparing the yield point and the ultimate strength we found that the yield point was more sensitive to the orientation parameter. This may be due to the reason previously suggested by Torres-Rendon *et al*.^20^ that the ultimate tensile strength is limited by defects excessively present in size or quantity in the structure and hence cannot be increased by solely enhancing orientation.

**Figure 7:**
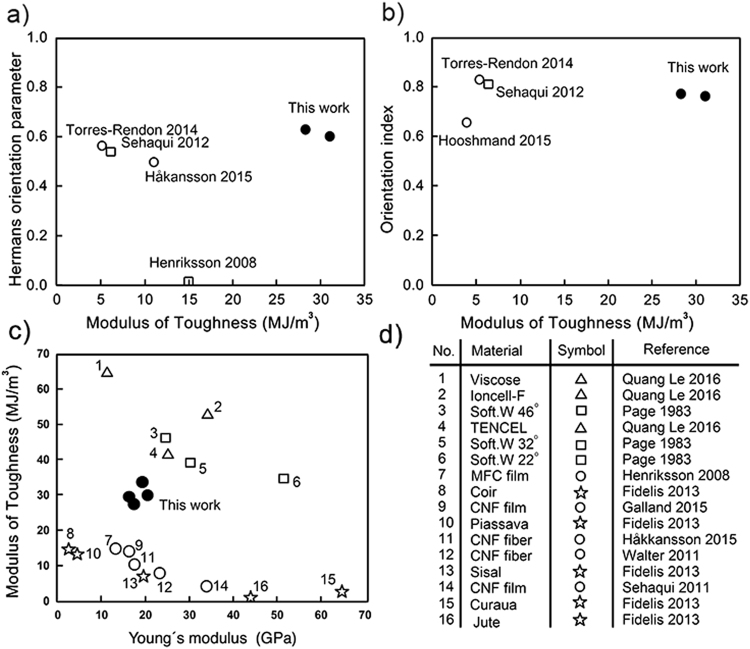
Orientation parameters vs. modulus of toughness. (**a**) Hermans orientation parameter and (**b**) orientation index plotted against the modulus of toughness. Degrees of orientation reached in this work (filled circles) were very similar to those of actively oriented fibers (empty circles) and films (empty squares) reported in the literature^9,12–14^. Note that the films by Sehaqui *et al*.^21^ were oriented by drawing before drying whereas those by Henriksson *et al*.^31^ were not drawn. Additionally, the shown values for orientation parameters of films are for the in-plane orientation. Both Hermans orientation and orientation index are plotted because both the values are not available for all relevant works. (**c**) and (**d**) Modulus of toughness vs. Young’s modulus for natural cellulosic fibers (empty squares and empty stars)^43,44^, regenerated cellulose based fibers (empty triangles)^47^, reported CNF based fibers from literature^6,17,19–21,31,32^ (empty circles) and this work (filled circles). Materials are numbered in order of decreasing toughness value.

Hermans orientation parameters and orientation indices from this work and the relevant literature are plotted against modulus of toughness (Fig. 7), and Young’s modulus (Supplementary Fig. S10). The comparison reveals that the degrees of orientation are very similar to those in related works where orientation was actively enhanced^17,20,21,31^. Further it can be concluded that, with the exception of the current work, a high orientation *per se* does not lead to a high modulus of toughness. This can be understood in the light of the typically observed reduction in extensibility upon increase of orientation, which subsequently leads to higher stiffness and strength. Interestingly, in the work by Henriksson *et al*.^31^ the orientation of the fibrils was low, yet still they reached a modulus of toughness significantly higher than those reported for much more oriented structures. Note that the orientation values for the films are of the in-plane orientation and do not take into account the high degree of out-of-plane orientation.

Within the experiments of this study, an increased alignment is experimentally consistent with the enhancement of all mechanical properties, including the modulus of toughness. However, it is interesting to consider why other studies in the literature reporting similar parameters of orientation, did not reach the same high toughness. Firstly, we recognize that obtaining a high modulus of toughness with increased stiffness and strength depends mostly on simultaneously retaining a high ultimate strain, given the typical shape of the stress-strain curve for CNF-based films and fibers. Stiffness, as well as strength, are from previous studies known to correlate directly with orientation^9,12–14^, but while enhancing these properties a simultaneous reduction in extensibility is often observed (Supplementary Table S3). Hence, the question arises as to why the ultimate strain was not reduced in this work as compared to previous studies. We note two hypotheses that our results may imply. One hypothesis relates to the fact that in comparable studies the orientation was induced only after or simultaneously as aggregation / coagulation was induced^16,20,21^. The second hypothesis relates to the material composition, the CNF used in this work was chemically unmodified and contains a significant fraction of hemicellulose.

Related to the first hypothesis, we note that in this study the alignment was induced in the capillaries, in a processing step well before the final coagulation. Previously, short extrusion nozzles have been used, and alignment has been pursued by stretching after extruding to a coagulation bath, or alternatively flow-focusing devices have been used to aggregate the CNFs by elevated ionic strength while simultaneously aligning by hydrodynamic pinching of the dispersion^6,9,12–14,17,19–21,31,32,43,44^. In such cases, typically higher strength and moduli lead to reduced strain and modulus of toughness. As schematically illustrated in Supplementary Fig. S11, we speculate that the deformation of the CNF network during or after coagulation may lead to the formation of poorly connected regions in the network when the fibrils slide excessively past each other and are no longer able to bridge adjacent regions. Such poorly connected regions will be expressed as structural defects in the final structure upon drying, and can be expected to cause premature failure of the material.

Related to the second hypothesis, the material composition, we note that the CNF used in this work contained a relatively large amount (24%) of hemicellulose. In the literature there are reports linking the function of hemicellulose to the toughness of cell wall structures based on its function as an adhesive matrix between cellulose fibrils^45^. Quantifying this effect experimentally in a setup as described in this work by for example enzymatic removal of hemicellulose is challenging because it is known that removing or modifying the hemicellulose fraction in CNF directly affects its rheological properties by making CNF more difficult do disperse, and leading to curling up of individual fibrlis^46^. As a result, the flow of CNF in the capillary is affected, making direct comparisons unfeasible.

Although strength is often desirable, practical requirements for technical materials are typically more diverse. The obtained CNF based fibers in this study illustrated significant property combinations, manifesting as high modulus of toughness vs. high stiffness comparing to any other reported CNF based fiber/film^6,17,19–21,31,32^ and many natural cellulosic fibers^43^ (Fig. 7c,d). Although still the fibers in this work show lower stiffness and toughness compared to high-density regenerated cellulose based fibers such viscose, TENCEL and Ioncel-F^44,47^ interestingly, these optimized materials also demonstrate a similar trend of mutually exclusive mechanical properties within their respective groups.

## Conclusions

We showed that we can attain remarkable high modulus of toughness *vs*. Young’s modulus or yield strength, by spinning of CNF fibers using long and thin extrusion capillaries and high flow rates, which caused the CNF to orient prior to coagulation. Solely the increase in alignment led to simultaneous increase in both stiffness and yield strength by roughly 50%. In stark contrast to the literature, the enhanced stiffness and yield strength were not achieved at the expense of ultimate strain, which remained in the range of 10–12% in all cases with 2% w/v CNF. Therefore, also substantially higher moduli of toughness (up to 31 MJ/m^3^) were obtained than previously reported. In the search for an understanding of toughness vs. strength relations^8^, and towards exploiting the full mechanical potential of CNF in spun fibers, it is hypothesized that not only the high alignment of the fibrils should be aimed at, but additionally minimization of the size and amount of structural defects, where the role of the processing conditions as well as surface interactions should receive attention.

## Materials and Methods

### CNF preparation

The CNF was prepared as previously described^48^. Briefly, never dried birch pulp (with 24% hemicellulose content) was disintegrated with a fluidizer (Microfluidics Corp., Newton, MA, USA.), giving a hydrogel with a consistency of approximately 1.63% w/v in water. To make homogenous and non-flocculated CNF dispersions above 2.5% w/v, we used mild stepwise centrifugation and homogenization.

### Characterization of CNF and extruded fibers

Atomic force microscopy (AFM) was used to study the morphology of CNF fibrils dried from dispersion (Fig. 1c). CNF samples were diluted 1:1000 with water and a 30 μL aliquot was spread on a freshly cleaved mica surface. Samples were allowed to dry for 24 hours at ambient temperature before the imaging. A Veeco dimension 5000 AFM instrument was used and images were recorded in tapping mode in air with scan rates of 0.8–1 Hz with a FASTSCAN-B cantilever.

### Polarized Optical Microscopy

Polarized optical microscopy (POM) imaging was used to qualitatively study birefringence of the fibers. Samples were placed between two cross-polarizers in an optical microscope (Leica DM4500 P LED) and interference color angles were determined at ±45°.

### Scanning electron microscopy

Scanning electron microscopy (SEM) imaging was performed with a Zeiss FE-SEM field emission microscope with variable pressure, operating at 1–1.5 kV for all the samples. A thin platinum coating was sputtered onto the samples prior to imaging of surfaces and cross-sections of the fibers.

### Rheological measurement

The rheological behavior of CNF at different concentrations was determined using a stress controlled rheometer (Modular Compact Rheometer, Model MCR 300) having a serrated plate geometry (diameter 22 mm). Measurements were carried out at 23 °C.

### Fiber spinning

For spinning of continuous fibers, an experimental setup was used consisting of four main parts; a pump, sample container, extrusion capillaries with different lengths and diameters, and a coagulation bath with ethanol (see Fig. 1). A high performance pump was used with fine adjustment of the flow rate to deliver eluent to a 10 ml sample container with a funnel shape outlet (10 mm in length with widest region of 5 mm and shortest region of 1mm), forcing the CNF to pass through the capillary that is connected to the outlet of the sample container. Extrusion capillaries with different lengths of 20, 200, and 1500 mm and internal diameters of 0.4, 0.5, 0.75, and 1.0 mm were used. Prior to use, the CNF was centrifuged (swing bucket rotor) for 60 min *at* 3000 RPM to remove air bubbles that would cause defects. CNF fibers were prepared by spinning of CNF dispersions through a capillary into a coagulation bath of 99.5% (v/v) ethanol. After 10 minutes in the bath, the fibers were removed and dried at ambient conditions with both ends fixed. The parameters systematically investigated were concentration of CNF, length and inner diameter of the extrusion capillaries and the flow rate.

### Mechanical characterization

The ends of the fibers were fixed by gluing them between two pieces of abrasive sandpaper. Tensile testing was performed on a 5 kN tensile/compression module (Kammrath & Weiss GmbH, Germany) using 100 N load cells. The elongation speed was 8.35 µm/second with a gauge length of 10 mm. Strain was calculated by dividing the absolute elongation with the original gauge length and multiplied by 100%. Before testing, the fibers were stabilized at 50% relative humidity for at least for 24 hours. Data were processed using Matlab (MathWorks) to extract the mean values and standard deviations for Young’s modulus, yield strength, slope after the yield strength, maximum strength, maximum strain and modulus of toughness (i.e. the area under the stress-strain curve). The ultimate strength was taken as the highest stress reached by each sample. The Young’s modulus was defined as the average slope of the stress-strain curve in the elastic region before the yield point. The yield point was defined as the point of intersection of the two first degree polynomials fitted to the linear regions of the elastic region and the beginning of the plastic deformation after the yield point. Cross-sectional areas of each fiber was determined form SEM images. The software package ImageJ^49^ was used for image processing. In cases where the cross section was not completely circular, a contour line was drawn to delineate the cross-sections.

### Wide angle X-ray scattering (WAXS)

Wide angle X-ray diffraction experiments were carried out at the µSpot beamline at BESSY II (Berliner Elektronenspeicherring-Gesellschaft für Synchrotronstrahlung, Helmholtz-Zentrum Berlin, Germany). The energy exposure used was 15 keV using a silicon 111 monochromator and a beam size of 100 µm. Fibers were clamped in a custom made tensile tester, which was mounted on a motorized stage, enabling positioning of the fibers perpendicular to the beam path. Diffractograms from several points along the fibers were recorded.

After subtraction of air scattering from the diffractograms, azimuthal intensity profiles at the (004) reflection were extracted by sector-wise integration after masking the diffractogram to show only the (004) reflection ring. From the thus obtained azimuthal intensity profile a constant baseline was reduced so that the value at 90° from the azimuthal maxima was set to zero. Subsequently, from the azimuthal intensity profile, the Hermans orientation parameter was calculated according to equations 1 and 2 and the orientation index (П) according to equation 3 ^21,50^.

1 $${\boldsymbol{S}}=\frac{3}{2}\langle {\bf{co}}{{\bf{s}}}^{{\boldsymbol{2}}}{\boldsymbol{\Phi }}\rangle -\frac{1}{2}$$

2 $$\langle {\bf{co}}{{\bf{s}}}^{{\boldsymbol{2}}}{\boldsymbol{\Phi }}\rangle =\frac{{\sum }_{{\boldsymbol{0}}}^{{\boldsymbol{\pi }}}{\boldsymbol{I}}({\boldsymbol{\Phi }})\sin \,{\boldsymbol{\Phi }}{\bf{co}}{{\bf{s}}}^{{\boldsymbol{2}}}{\boldsymbol{\Phi }}}{{{{\boldsymbol{\sum }}}^{}}^{{\boldsymbol{\pi }}}{\boldsymbol{I}}({\boldsymbol{\Phi }})\sin \,{\boldsymbol{\Phi }}}$$

3 $${\boldsymbol{\Pi}}=\frac{{\bf{180}}-{\boldsymbol{FWHM}}}{{\bf{180}}}$$

where FWHM is the Full-Width-at-Half-Maximum.

## Electronic supplementary material

Supplementary Information
