# Supplementary material for: Aligning cellulose nanofibril dispersions for tougher fibers
Source: Sci Rep. 2017 Sep 19;7:11860. doi: 10.1038/s41598-017-12107-x (PMC5605715; doi:10.1038/s41598-017-12107-x)
Supplement: Supplementary file 1 — Supplementary Information [file 41598_2017_12107_MOESM1_ESM.doc]

Supplementary Information

**Aligning cellulose nanofibril dispersions for tougher fibers**

Pezhman Mohammadi^1,+^, Matti S. Toivonen^2,+^, Olli Ikkala^2^, Wolfgang Wagermaier^3^, and Markus B. Linder^1^*

^1^ Department of Bioproducts and Biosystems, School of Chemical Engineering, Aalto University, P.O. Box 16100, FI-16100, Espoo, Finland.

^2^ Department of Applied Physics, School of Science, Aalto University, P.O. Box 15100, FI-00076, Espoo, Finland.

^3^ Department of Biomaterials, Max Planck Institute of Colloids and Interfaces, D-14424 Potsdam, Germany.


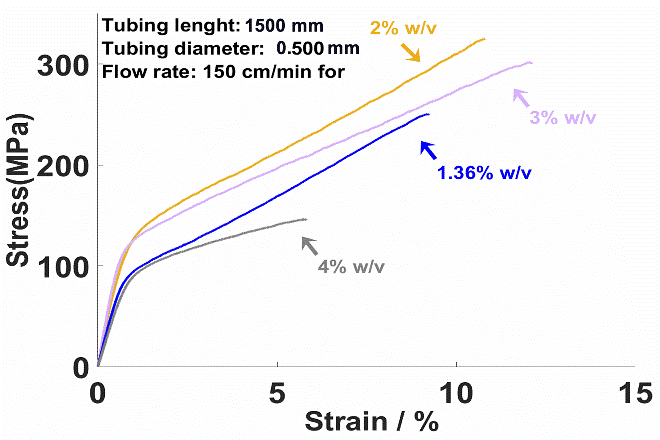


**Figure S1**. Representative stress-strain curves for the fibers made from varying the CNF aqueous concentration of 1.36% w/v, 2% w/v and 3% w/v, extruded using a 1500 mm/0.5 mm (length/inner diameter) capillary with the linear flow rate of 150 cm/min. 1.36 % w/v was the lowest CNF concentration that resulted in fibers after removing water in the ethanol coagulation bath and drying. All CNF suspensions below 2.5 % w/v were homogenous and without visible aggregation, and led to stable spinning. CNF above 3.75% w/v did not result in stable fibers, and concentrations above 4 % w/v were not feasible because of excessive back-pressure formation in the device. Therefore, four different concentrations of CNF dispersions (1.36, 2, 3, and 4 % w/v) were selected for further analysis.

For the concentration 4% w/v CNF, only short fibers of poor quality could be achieved, and the stress-strain curve was excluded from the dataset. Instead the curve shown is for a shorter and larger capillary (200 mm/0.75 mm) and a lower flow rate (50 cm/min). Mean values and standard deviation (N=30) for mechanical properties of the fibers spun with different concentrations of CNF are shown in Table S1. Fibers made from 2 and 3 % w/v aq. dispersion of CNF gave ultimate tensile strengths of above 300 MPa and Young’s moduli close to 17 GPa, while fibers made from 1.36 % w/v CNF gave a lower ultimate tensile strength of 258 MPa and a Young’s modulus of 12 GPa. The effect of concentration on the yield stress was another prominent feature, where concentrations of 2 and 3 % w/v rendered 139 MPa and 118 MPa, respectively, in comparison to 86 MPa for the concentration of 1.36 % w/v. Fibers made from 2 % w/v and 3 % w/v CNF showed high strain-to-failure values of around 12%. 2 % w/v was selected as the CNF concentration for further experiments.


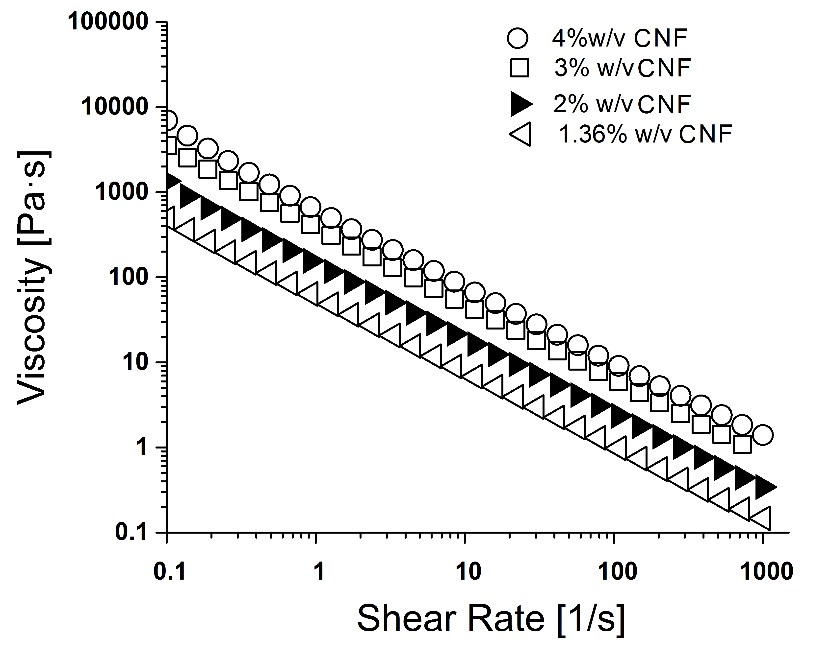


**Figure S2**. Shear thinning of CNF. Viscosity was measured for four different concentrations of aq. CNF dispersion. At the low shear rate (0.1 s^-1^), the viscosity increased from 473, 1350, 3630, to 6970 Pa.s by increasing the CNF aq. concentrations from 1.4, 2, 3, to 4 % w/v, respectively. All samples showed considerable shear thinning.


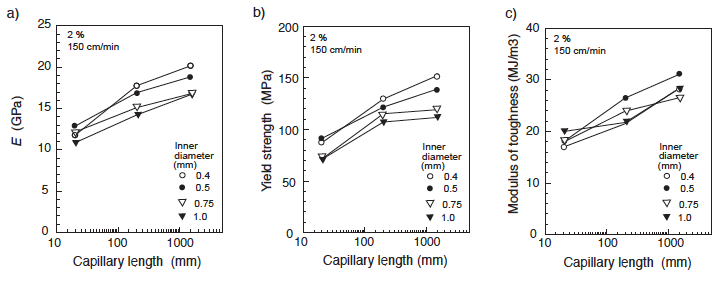


**Figure S3**. The mean values for the Young’s modulus, yield stress, and modulus of toughness as a function of the capillary length and dimeter. As can be seen by increasing the capillary length, all the aforementioned mechanical properties increased for the optimum CNF concentration (2 w/v) using high flow rate (150 cm/min). Reducing the dimeter of capillary also enhances the Young’s modulus, yield stress, and modulus of toughness but the effect of the length of the capillary is more prominent.


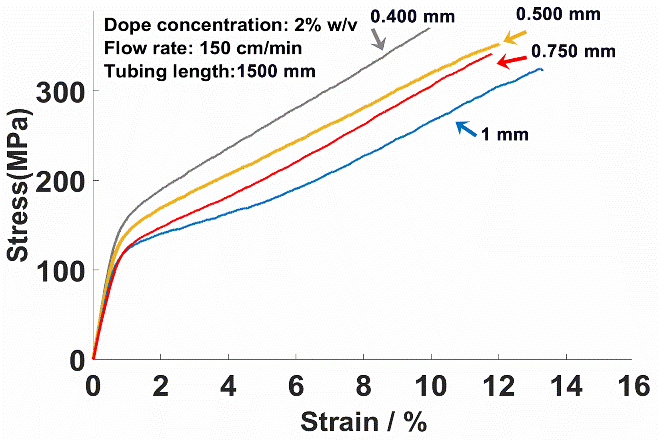


**Figure S4**.The effect of diameter in the mechanical properties was also significant, but less pronounced than the effect of the capillary length. With a decrease in diameter (from 1.0 mm to 0.4 mm) while keeping the length at 1500 mm, we observed an increase in both Young’s modulus and yield strength. These changes correspond to increases of 21 % and 36 % in Young’s modulus and yield strength, respectively.


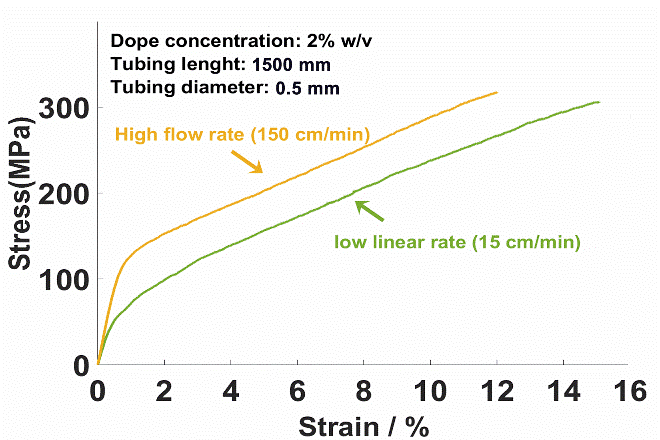


**Figure S5**. To study the effect of linear flow rate (i.e. shear rate of the CNF dispersion in the capillary), high and low flow rates were selected; 150 and 15 cm/min, respectively (detailed data is tabulated in Table S2, (N=30)). Fibers spun with the higher linear flow rate showed higher tensile strength, yield strength and Young’s modulus (328 MPa, 139 MPa and 18.84 GPa, respectively), in comparison to the fibers extruded with the lower flow rate (296 MPa, 78 MPa and 12.6 GPa, respectively). Interestingly, the effect of high linear flow rate on mechanical properties was more pronounced with longer and thinner capillaries (Table SI2). In contrast, while using short capillaries and high flow rate changing the diameter of capillary had only a small effect on the mechanical properties. Also, for a low flow rate, the yield point becomes less prominent. One can conclude that higher flow rate improves the mechanical properties and allows a more pronounced yield point. Therefore, the higher flow rate was selected for subsequent investigations.


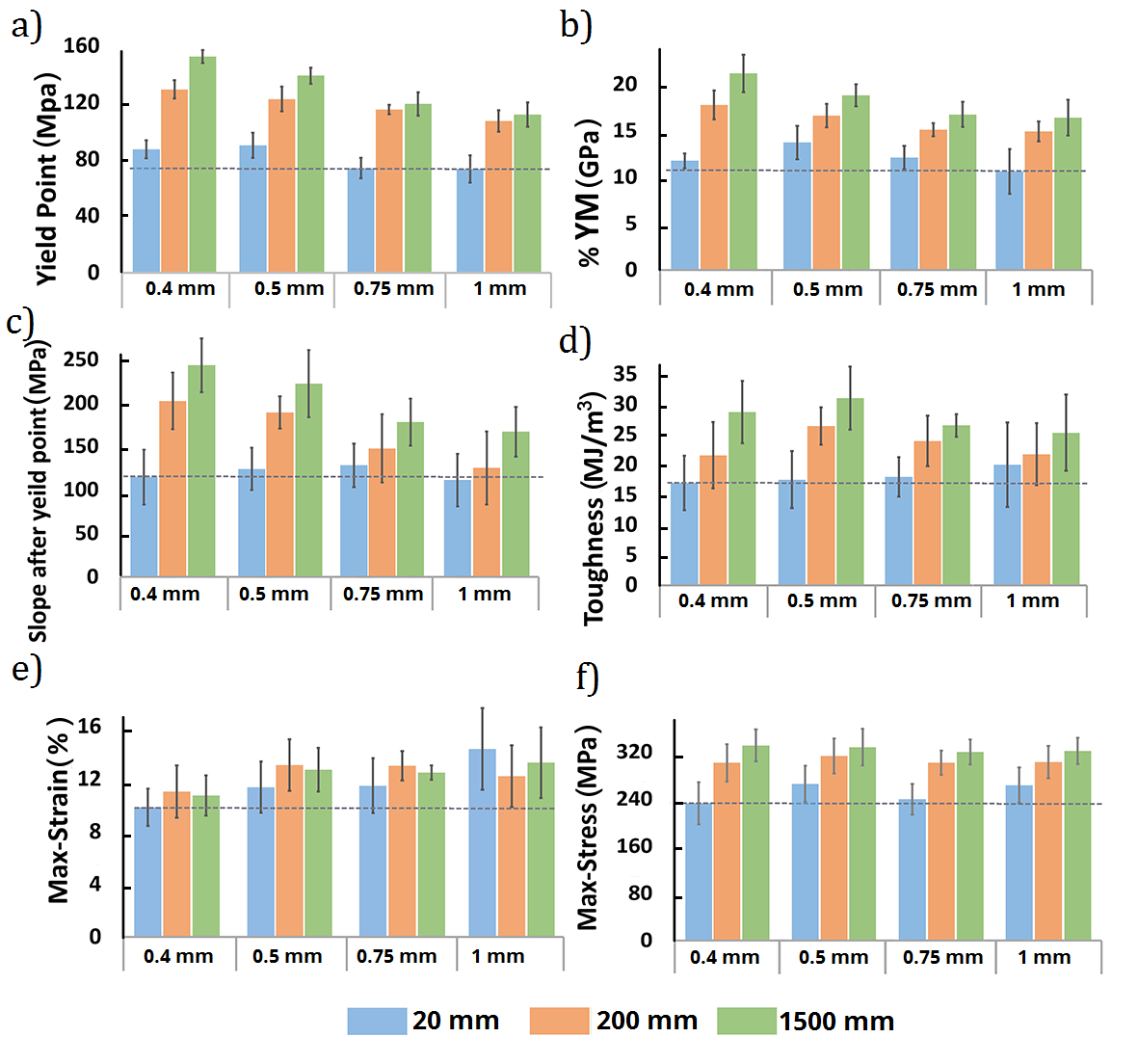


**Figure S6**. Mean value and standard deviations of mechanical properties of the fibers spun using different lengths and dimeters of the spinning capillary. a) Yield point, b) young’s modulus c) slope after the yield point, d) toughness, e) Maximum strain and f) Max-stress. In all the graphs, blue bar (20 mm capillary), orange bar (200 mm capillary) and green bar (1500 mm capillary).


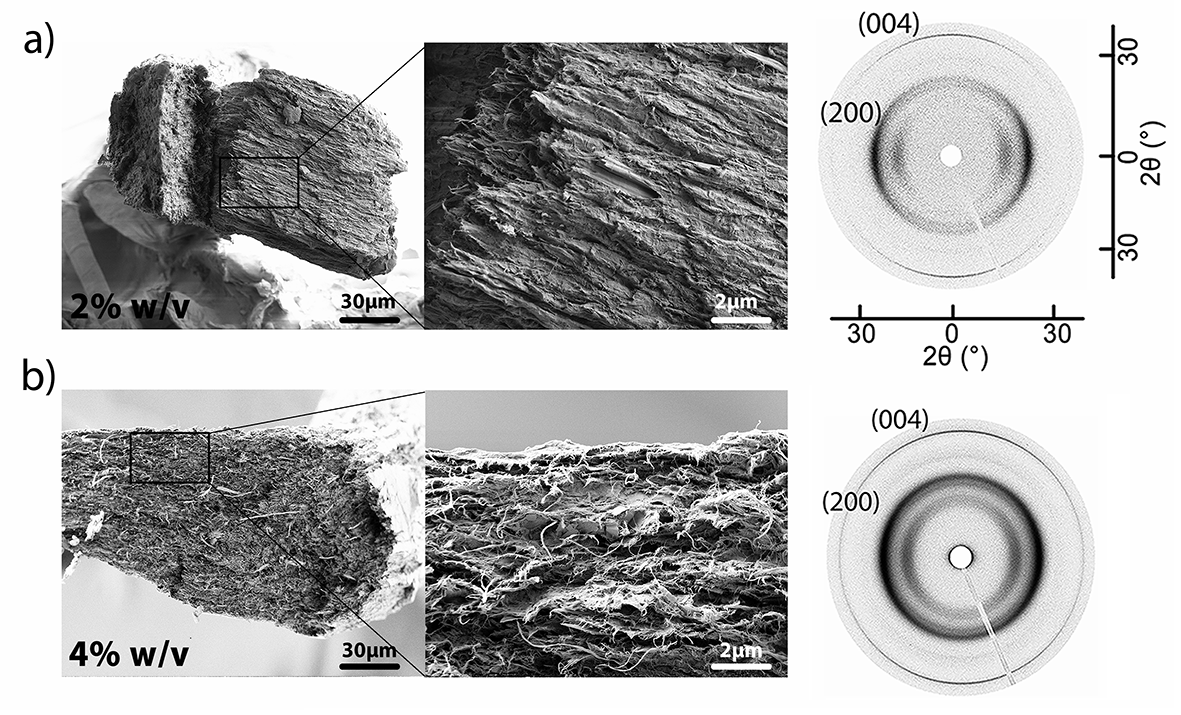


**Figure S7**. SEM micrographs and wide angle X-ray diffraction of single fiber of cross section of freeze fractured fibers. a) 2% w/v aq. CNF concentration. b) 4% w/v aq. CNF concentration. The flow rate used for extrusion of 2% w/v fibers were 150 cm/min, 1500 mm/0.5mm. For 4% w/v a shorter capillary, larger diameter (20 mm/0.75 mm) and a lower flow rate (50 cm/min) were used.


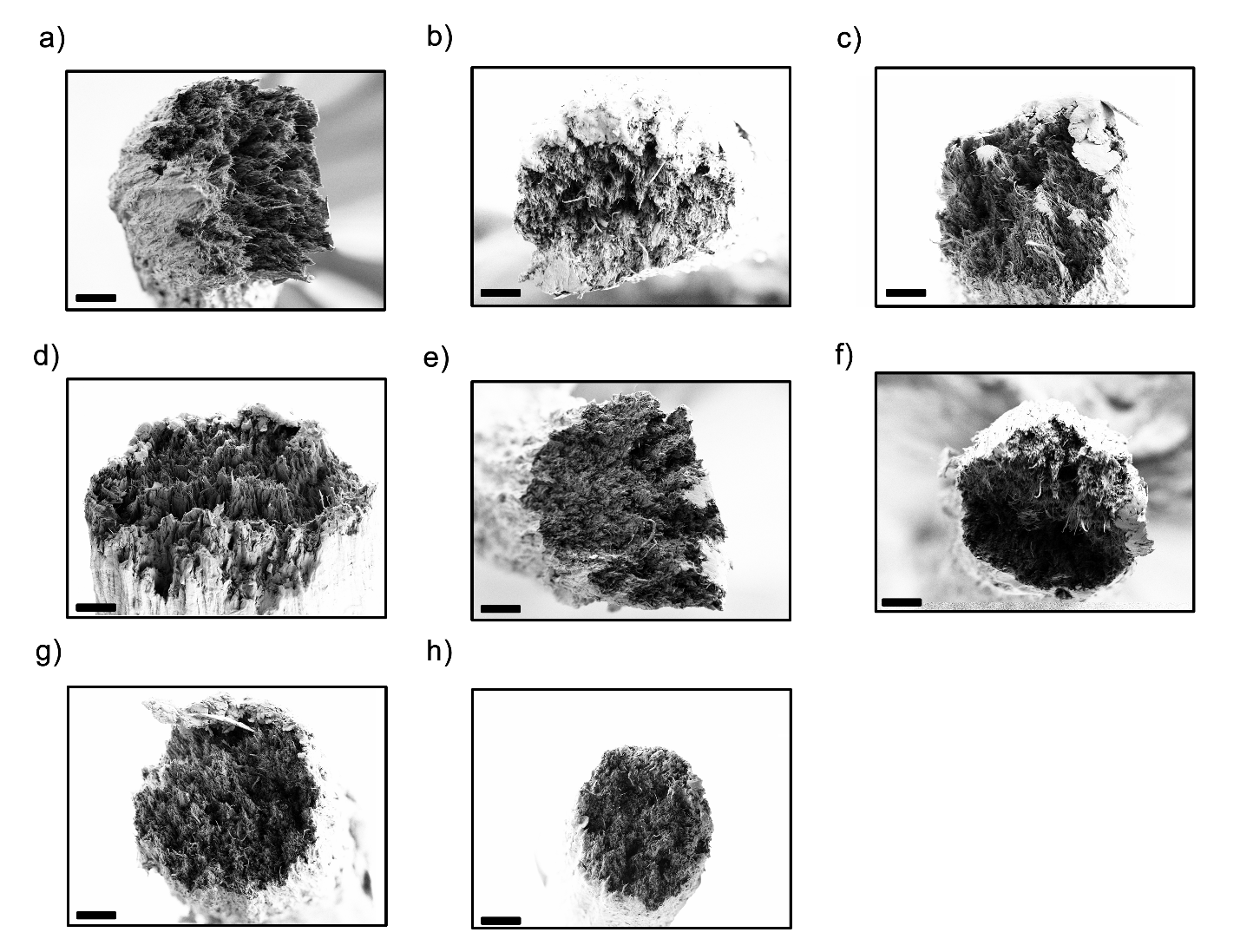


**Figure S8.** SEM images of cross sections of fibers after tensile testing. The fractured ends for all fibers were blunt, with no visible evidence of major defects. The morphology between samples was similar and the differences between the degree of orientation in the fibers did not lead to visible differences in the fractured surfaces. a) Fiber spun with 200 mm/0.75 mm-4 % w/v-50 cm/min spinning parameter, b) fiber spun with 20 mm/0.5 mm-2% w/v-150 spinning parameter, c) Fiber spun with 1500 mm/0.5 mm-2% w/v-15 cm/min spinning parameter, d) Fiber spun with 1500 mm/1.0 mm-2% w/v-150 cm/min spinning parameter, e) Fiber spun with 1500 mm/0.75 mm-2% w/v-150 cm/min spinning parameter, f) Fiber spun with 200 mm/0.5 mm–2% w/v-150 cm/min spinning parameter, g) Fiber spun with 1500 mm/0.5 mm-2% w/v-150 cm/min spinning parameter and h) Fiber spun with 1500 mm/0.4 mm-2% w/v-150 cm/min spinning parameter (Scale bars are 30 µm).


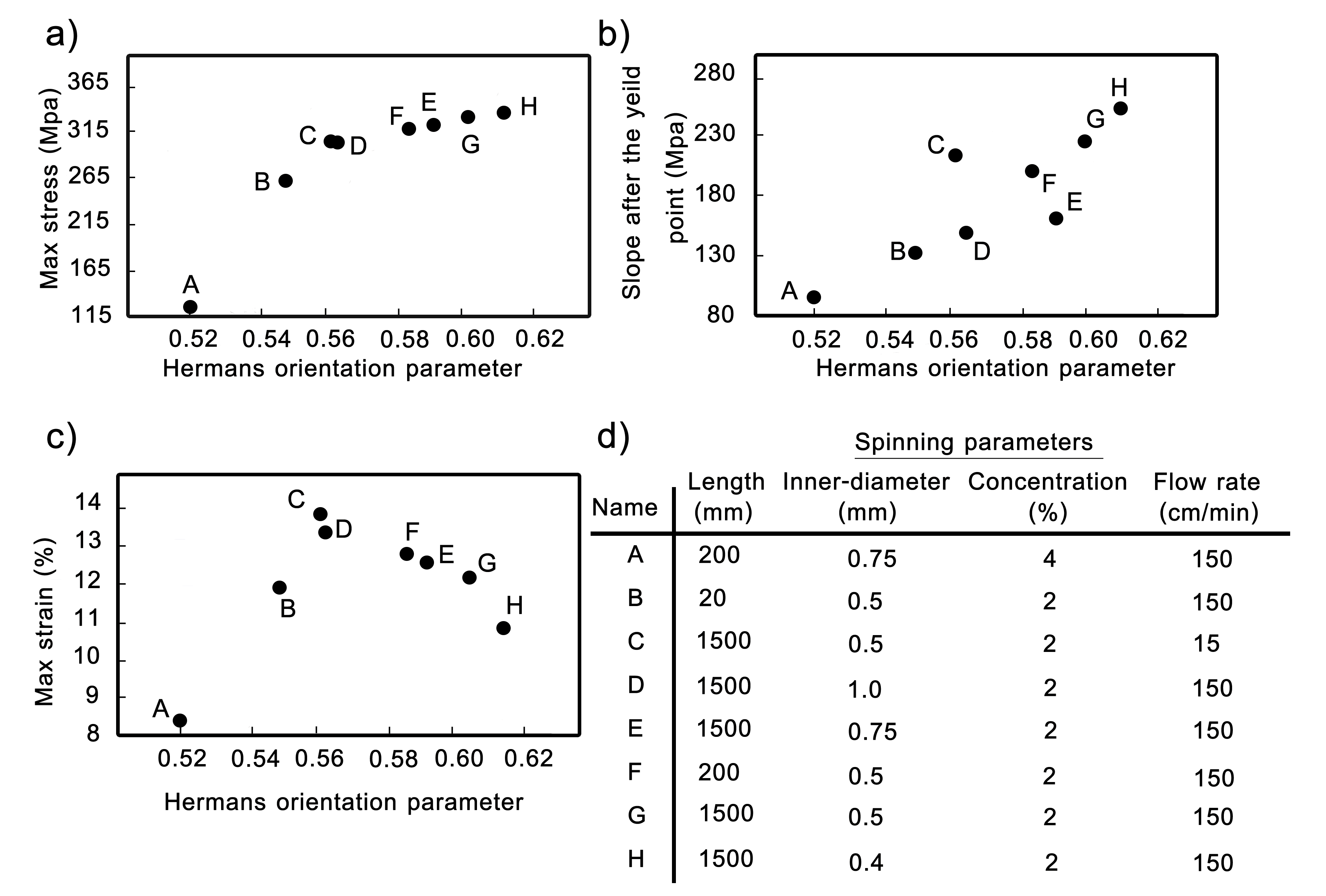


**Figure S9**. Hermans orientation parameter calculated from the (004) reflection *vs.* a) the ultimate tensile strength, b) the slope after the yield point, and c) ultimate strain for fibers spun at high and low flow rates with different lengths and diameters of spinning tubings and concentration of CNF suspension. Sample specific spinning parameters are summarized in d).


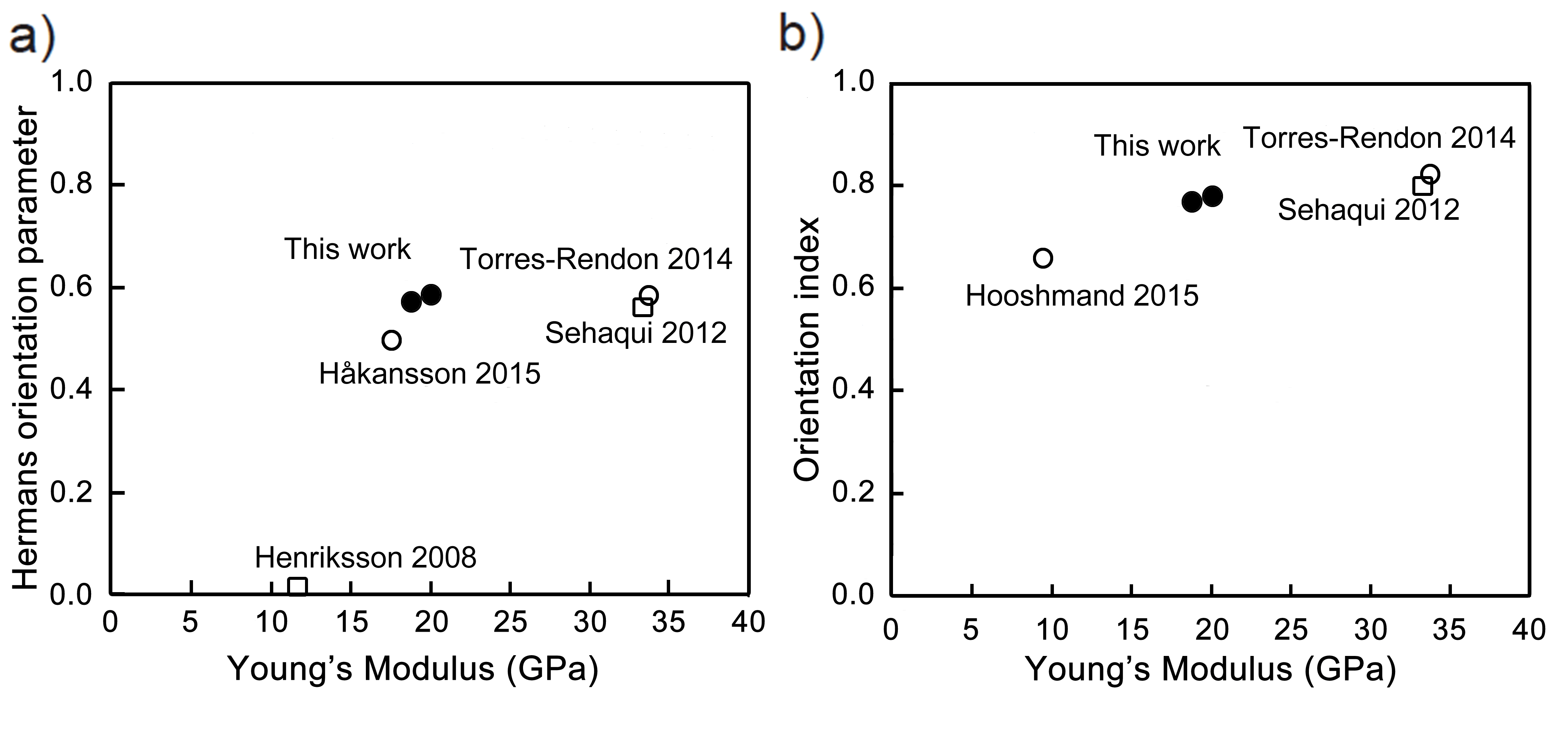


**Figure S10. Orientation parameters *vs.* Young’s moudulus.** a) Hermans orientation parameter and b) orientation index plotted *vs.* Young’s modulus. This work marked with black solid circle. Comparable data from related works draw with open symbols; films with squares and fibers with circles.


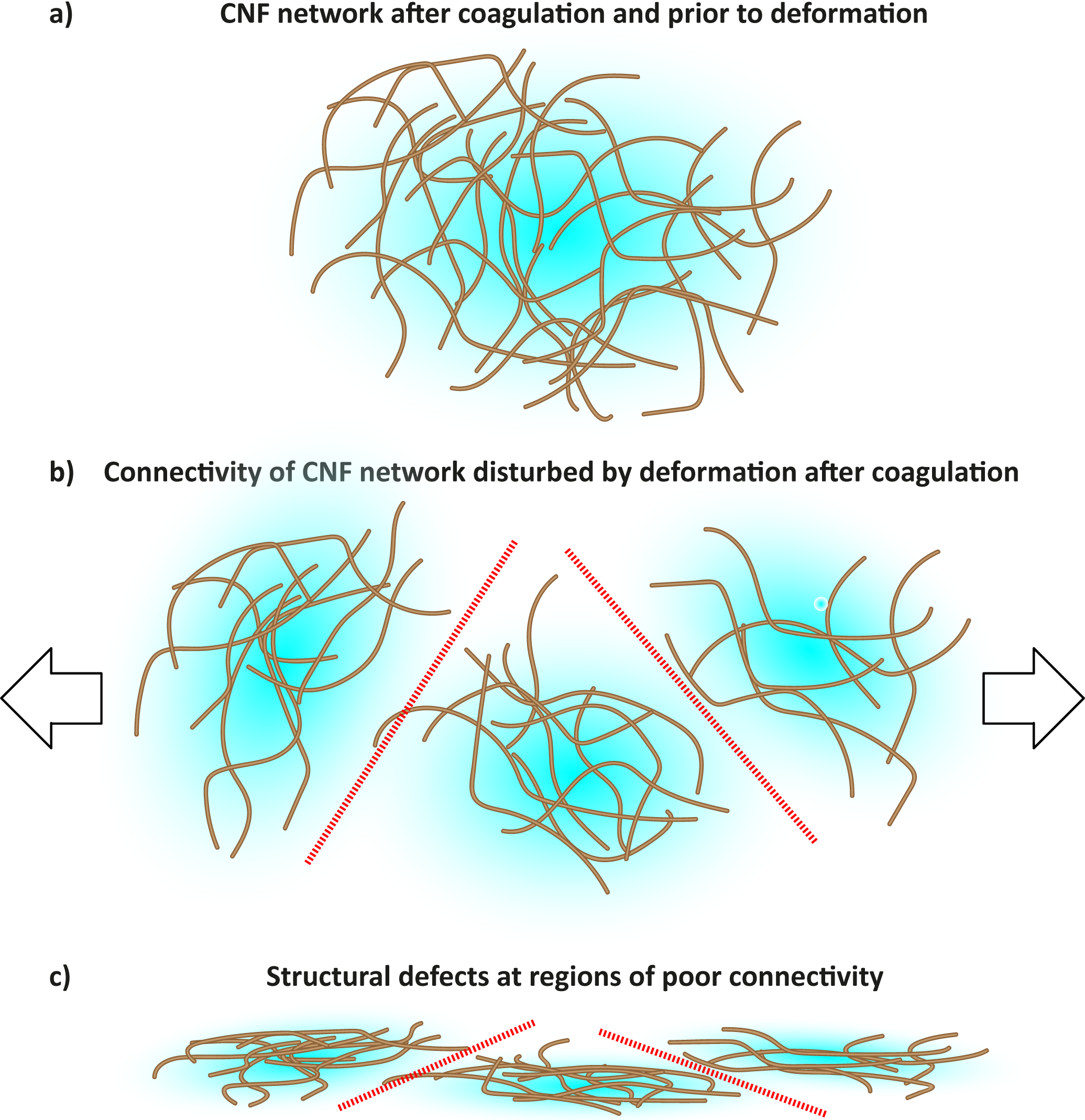


**Figure S11. Schematic illustration of the hypothesized mechanism for formation of structural defects upon deformation of the CNF network after coagulation.** a) Prior to deformation the coagulated CNF network is homogeneous and all regions are well connected. b) When the network is deformed, the fibrils slide past each other and separate to flocs which are poorly bridged by fibrils penetrating into adjacent flocs, as highlighted with the red dashed line. (c) Upon drying of the CNF network the areas of low connectivity are present in the final structure, and will act as weak points that fail first upon deformation.

| **Table S1.** Summary of mechanical properties of fibers produced with different CNF concentrations. 30 samples were characterized from each batch. | | | | |
| --- | --- | --- | --- | --- |
| Dimensions of extrusion capillary (length / inner diameter) | 1500 mm/0.5 mm | 1500 mm/0.5 mm | 1500 mm/0.5 mm | 200 mm/0.75 mm |
| Concentration [w/v] | 1.36 % w/v | 2 % w/v | 3 % w/v | 4 % w/v |
| Flow rate (cm/min) | 150 | 150 | 150 | 50 |
| Name | --- | G | --- | A |
| Diameter (µm) | 41.82 ±5 | 41.62 ±7 | 62.18 ±12 | 117.37 ±34 |
| Strength [MPa] | 256 ±35.68 | 328 ±40.28 | 304 ±20.97 | 127 ±68.11 |
| Extensibility [%] | 9.87 ±1.41 | 12.10 ±3.65 | 11.94. ±1.07 | 8.35 ±5.11 |
| Young’s modulus [GPa] | 10.18 ±1.39 | 18.84 ±1.52 | 15.16 ±1.97 | 11.86 ±9.41 |
| Yield Point [MPa] | 86.37 ±6.082 | 139.04 ±8.66 | 118 ±10.69 | 76.69 ±19.44 |
| Slope after yield point [MPa] | 188.77 ±23.82 | 234.37 ±36.68 | 180 ±32.33 | 95.80 ±61.23 |
| Toughness [MЈ/m^-3^] | 15.98 ±4.51 | 31.03 ±7.23 | 16.11 ± 3.26 | 8.98 ± 16.01 |
|  | | | | |

**Table S1**. Summary of mechanical properties as dependent on concentration of extruded CNF suspension and spinning parameters. Four different concentration of CNF suspensions were tested (1.36% w/v, 2% w/v, 3% w/v and 4% w/v)

**Table S2**. The effect of flow on mechanical parameters under different conditions. The values are along with other combinations of the capillary dimensions, also showing standard deviations (N=30). Fibers extruded from longer capillaries (200 mm/0.5 mm and 1500 mm/0.5 mm) presented tensile strengths over 300 MPa, Young’s moduli above 14.2 GPa and values for moduli of toughness higher than 21 MJ/m^3^. Tensile strength, Young’s modulus and toughness of fibers increased by 13 %, 11% and 17 %, respectively, upon using the long 1500 mm/0.5 mm capillary instead of the short 20 mm/0.5 mm capillary. For comparison, upon extrusion from 20 mm/0.5 mm capillaries they were lower 265 MPa, 12.8 GPa and 17.9 MJ/m^3^, respectively. The values observed for capillaries with 1500 mm/1.0 mm were 16.6 GPa and 111.5 MPa, while those for capillaries with 1500 mm/0.4 mm were 20.1 GPa and 152 MPa, respectively.

| **Table S2.** Summary of Mechanical properties of filaments at high and low flow rates with different lengths and diameters of spinning tubings spun with 2% w/v optimum dope concentration. (Number of repeats: 30) | | | | | | | | | |
| --- | --- | --- | --- | --- | --- | --- | --- | --- | --- |
| Tubing dimension | 20 mm/0.4 mm | | | 20 mm/0.5 mm | | 20 mm/0.75 mm | | 20 mm/1.0 mm | |
| Concentration [w/v] | 2% w/v | | | 2% w/v | | 2% w/v | | 2% w/v | |
| Flow rate (cm/min] | 150 | | 15 | 150 | 15 | 150 | 15 | 150 | 15 |
| Name | --- | | --- | **B** | --- | --- | --- | --- | --- |
| Diameter (µm) | 40.73 ±8 | | 43.60 ±10 | 58.85 ±7 | 53.14 ±4 | 99.80 ±10 | 97.23 ±9 | 114.02 ±15 | 111.96 ±14 |
| Strength [MPa] | 236 ±35.68 | | 215 ±35.79 | 265 ±30.52 | 244 ±27.47 | 241 ±12.97 | 257 ±16.58 | 263 ±30.01 | 251 ±24.77 |
| Extensibility [%] | 9.87 ±1.41 | | 11.87 ±3.56 | 11.64 ±1.90 | 12.34 ±2.82 | 11.53 ±2.08 | 13.32 ±3.76 | 14.35 ±3.13 | 13.83 ±4.14 |
| Young’s Modulus [GPa] | 11.77 ±0.79 | | 12.31 ±1.27 | 12.87 ±1.81 | 11.91 ±2.12 | 12.16 ±0.86 | 10.19 ±1.30 | 10.86 ±1.41 | 9.35 ±1.82 |
| Yield stress [MPa] | 87.03 ±15.32 | | 61.75 ±21.64 | 91.24 ±15.78 | 66.64 ±20.63 | 71.27 ±16.21 | 69.45 ±18.74 | 70.92 ±17.67 | 64.01 ±25.08 |
| Slope after yield point [MPa] | 118 ±31.75 | | 110.13 ±17.65 | 128.31 ±19.23 | 125.85 ±10.65 | 139.54 ±25.10 | 129.19 ±29.65 | 108.53 ±17.27 | 125.42 ±20.54 |
| Toughness [MЈ/m^-3^] | 16.97 ±4.51 | | 14.99 ±5.19 | 17.99 ±4.71 | 13.62 ±4.42 | 17.92 ±3.25 | 18.73 ±6.10 | 19.98 ±7.06 | 18.01 ±5.03 |
|  | | | | | | | | | |
| Tubing dimension | 200 mm/0.4 mm | | | 200 mm/0.5 mm | | 200 mm/0.75 mm | | 200 mm/1.0 mm | |
| Concentration [w/v] | 2% w/v | | | 2% w/v | | 2% w/v | | 2% w/v | |
| Flow rate (cm/min] | 150 | 15 | | 150 | 15 | 150 | 15 | 150 | 15 |
| Name | --- | --- | | **F** | --- | --- | --- | --- | --- |
| Diameter (µm) | 37.40 ±7 | 34.70 ±8 | | 46.07 ±9 | 47.54 ±7 | 82.64 ±10 | 86.04 ±9 | 112.12 ±12 | 110.65 ±17 |
| Strength [MPa] | 301 ±31.66 | 297 ±40.83 | | 313 ±21.59 | 283 ± 37.19 | 302 ±20.64 | 300 ±44.12 | 305 ±29.30 | 310 ±19.26 |
| Extensibility [%] | 11.04 ±1.99 | 12.33 ±2.59 | | 13.05 ±1.96 | 11 ± 1.70 | 13.34 ±1.37 | 13.27 ±3.10 | 12.26 ±2.68 | 11.72 ±3.30 |
| Young’s Modulus [GPa] | 17.76 ±1.54 | 12.91 ±3.70 | | 16.92 ±0.74 | 12.53 ±2.22 | 15.11 ±0.69 | 11.57 ±1.23 | 14.22 ±1.08 | 10.13 ±3.01 |
| Yield stress [MPa] | 129.40 ±6.48 | 76.23 ±13.45 | | 121.59 ±8.66 | 83.31 ±15.69 | 115.17 ±9.27 | 61.79 ±9.89 | 107,13 ±8.52 | 60.46±12.19 |
| Slope after yield point [MPa] | 218.08 ±32.63 | 157.82 ±28.53 | | 196.54 ±21.41 | 186.58 ±29.64 | 148.45 ±18.89 | 134.95 ±15.45 | 129.73 ±42.43 | 120.39 ±37.75 |
| Toughness [MЈ/m^-3^] | 21.48 ±5.49 | 16.50 ±10.54 | | 26.46 ±3.19 | 19.96 ±8.62 | 23.91 ±4.18 | 22.11 ±7.91 | 21.73 ±5.14 | 24.84 ±5.89 |
|  | | | | | | | | | |
| Tubing dimension | 1500 mm/0.4 mm | | | 1500 mm/0.5 mm | | 1500 mm/0.75 mm | | 1500 mm/1.0 mm | |
| Concentration [w/v] | 2% w/v | | | 2% w/v | | 2% w/v | | 2% w/v | |
| Flow rate (cm/min] | 150 | | 15 | 150 | 15 | 150 | 15 | 150 | 15 |
| Name in other figures / tables | **H** | | --- | **G** | **C** | **E** | --- | **D** | --- |
| Diameter (µm) | 35.49 ±5 | | 31.59 ±3 | 41.62 ±7 | 48.80 ±4 | 76.91 ±6 | 71.01 ±6 | 103.66 ±16 | 111.13 ±13 |
| Strength [MPa] | 331 ±34.27 | | 309 ± 30.57 | 328 ±40.28 | 296 ±29.33 | 316 ±12.97 | 287 ±38.11 | 305 ±19.30 | 297 ±34.57 |
| Extensibility [%] | 10.76 ±1.91 | | 12.8 ±2.1 | 12.10 ±2.65 | 12.44 ±1.89 | 12.38 ±1.77 | 14.31 ±3.2 | 13.26 ±2.88 | 18.1 ±5.96 |
| Young’s Modulus [GPa] | 20.17 ±2.02 | | 13.21 ±2.1 | 18.84 ±1.52 | 12.56 ±2.15 | 16.78 ±1.15 | 12.15 ±3.12 | 16.67 ±1.93 | 11.41 ±3.89 |
| Yield stress [MPa] | 152.06 ±9.57 | | 69.79 ±12.84 | 139.04 ±8.66 | 78.66 ±14.21 | 119.08 ±7.56 | 73.48 ±16.54 | 111.57 ±11.60 | 66.45 ±17.79 |
| Slope after yield point [MPa] | 250.80 ±45.74 | | 151.72 ±32.03 | 234.37 ±36.68 | 141.47 ±31.24 | 159.65 ±19.97 | 148.01 ±17.00 | 147.26 ±22.43 | 137.44 ±18.96 |
| Toughness [MЈ/m^-3^] | 28.31 ±5.14 | | 22 ±8.22 | 31.03 ±7.23 | 28.14 ±9.43 | 26.51 ±3.98 | 25.12 ±9.4 | 28.28 ±6.32 | 26.62 ±14.85 |
|  | | | | | | | | | |

| **Table S3. Summary of mechanical properties of CNF-based materials reported in the literature and those of two fibers reported in this work.** | | | | | | | | |
| --- | --- | --- | --- | --- | --- | --- | --- | --- |
| **Source** | **CNF Type** | **Film/Fiber** | **Post-stretch** | **Strength (MPa)** | **Strain**  **(%)** | **Yield Point (MPa)** | **Stiffness (GPa)** | **Toughness (MЈ/m^3^)­­** |
| **Henriksson *et al.* 2008** | Non-modified CNF | Film | No | 214 ± 7 | 10.1 ± 1.4 | 92.2 ± 5.2 | 13.2 ± 0.6 | 15 ± 1.9 |
| **Iwamoto *et al.* 2011** | TEMPO-CNF | Fiber | No | 321 ± 145 | 2.2 ± 1.2 | X^a^ | 23.6 ± 2.1 | X^a^ |
| **Sehaqui *et al.* 2011** | TEMPO-CNF | Film | Yes | 397 ± X^a^ | 1.8 ± X^a^ | ≃250^b^ | 33.3 ± X^a^ | ≃6 ^b^ |
| **Walther *et al.* 2011** | TEMPO-CNF | Fiber | Yes | 275 ± 15 | 4 ± 0.2 | ≃175^b^ | 22.5 ± 0.4 | 7.9 ± 0.8 |
| **Torres-Rendon *et al.* 2014** | TEMPO-CNF | Fiber | No | 289 ± 37 | 1.6 ± 0.3 | ≃160^b^ | 33.7 ± 4 | ≃5^b^ |
| **Håkansson *et al.* 2015** | Carboxymethylated CNF | Fiber | No | 490 ± 86 | 6.4 ± 1.6 | ≃210^b^ | 17.6 ± 0.7 | ≃11^b^ |
| **Galland *et al.* 2015** | Non-modified CNF | Film | No | 319 ± 9 | 7.1 ± 0.4 | ≃110^b^ | 16.2 ± 1 | ≃14^b^ |
| **Hooshmand *et al.* 2015** | Non-modified CNF | Fiber | No | 222 ± 16 | ≃3^b^ | ≃175^b^ | 12.6 ± 1.5 | ≃4^b^ |
| **This study (Fiber G)** | Non-modified CNF | Fiber | No | 328 ± 40 | 12.1 ± 2.3 | 139 ± 8.7 | 18.8 ± 1.5 | 31.0 ± 7.2 |
| **This study (Fiber H)** | Non-modified CNF | Fiber | No | 331 ± 34 | 10.8 ± 1.9 | 152.1 ± 9.5 | 20.2 ± 2.0 | 28.3 ± 5.1 |
| **a.No data in the publication**  **b.Values estimated from stress-strain curve in the publication.** | | | | | | | | |

**Table S3**. Comparison of mechanical properties achieved in the literature with focus on improving the mechanical properties by affecting the alignment of CNF in form of film and fibers or selection of material composition.

| **Table S4.**  Hermans orientation parameter, FWHM and orientation index calculated from (004) reflection from WAXS diffractograms | | | | | | | | |
| --- | --- | --- | --- | --- | --- | --- | --- | --- |
| **Fiber name** | **A** | **B** | **C** | **D** | **E** | **F** | **G** | **H** |
| Hermans orientation parameter | 0.5196 | 0.5584 | 0.5600 | 0.5677 | 0.5922 | 0.6038 | 0.5867 | 0.6137 |
| FWHM | 53 | 50 | 48 | 48 | 48 | 43 | 42 | 37 |
| Orientation index | 0.7056 | 0.7222 | 0.7333 | 0.7333 | 0.7333 | 0.7611 | 0.7667 | 0.7944 |

**Table S4**. Orientation parameters computed from WAXS diffractograms for single fibers spun at high and low flow rates with different lengths and inner diameters of extrusion capillaries spun with two different concentrations of CNF suspension. (A: 200 mm/0.75 mm-4 % w/v-50 cm/min, B: 20 mm/0.5 mm-2% w/v-150, C: 1500 mm/0.5 mm-2% w/v-15 cm/min, D: 1500 mm/1.0 mm-2% w/v-150 cm/min, E: 1500 mm/0.75 mm-2% w/v-150 cm/min, F: 200 mm/0.5 mm–2% w/v-150 cm/min, G: 1500 mm/0.5 mm-2% w/v-150 cm/min, H: 1500 mm/0.4 mm-2% w/v-150 cm/min (See details in Table S1 and S2))
